# Structure of the Mammalian Ribosome-Sec61 Complex to 3.4 Å Resolution

**DOI:** 10.1016/j.cell.2014.05.024

**Published:** 2014-06-19

**Authors:** Rebecca M. Voorhees, Israel S. Fernández, Sjors H.W. Scheres, Ramanujan S. Hegde

**Affiliations:** 1 MRC Laboratory of Molecular Biology, Francis Crick Avenue, Cambridge, CB2 0QH, UK

## Abstract

Cotranslational protein translocation is a universally conserved process for secretory and membrane protein biosynthesis. Nascent polypeptides emerging from a translating ribosome are either transported across or inserted into the membrane via the ribosome-bound Sec61 channel. Here, we report structures of a mammalian ribosome-Sec61 complex in both idle and translating states, determined to 3.4 and 3.9 Å resolution. The data sets permit building of a near-complete atomic model of the mammalian ribosome, visualization of A/P and P/E hybrid-state tRNAs, and analysis of a nascent polypeptide in the exit tunnel. Unprecedented chemical detail is observed for both the ribosome-Sec61 interaction and the conformational state of Sec61 upon ribosome binding. Comparison of the maps from idle and translating complexes suggests how conformational changes to the Sec61 channel could facilitate translocation of a secreted polypeptide. The high-resolution structure of the mammalian ribosome-Sec61 complex provides a valuable reference for future functional and structural studies.

## Highlights

• A near-complete atomic resolution structure of the mammalian ribosome
• Snapshot of a translating ribosome with hybrid state tRNAs and nascent polypeptide
• Structures of the Sec61 translocon bound to idle and translating ribosomes
• Molecular details of the residues involved in the ribosome-Sec61 interaction

## Graphical Abstract

**Figure:**
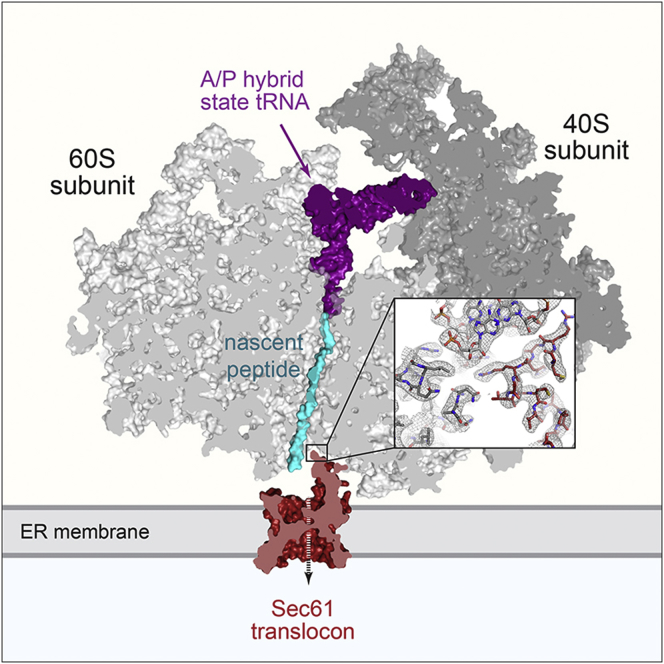

## Teaser

High-resolution structures of the cotranslational translocation complex in two states containing either an idle or actively translating ribosome provide an unprecedented view of the mammalian ribosome, the translocation channel, and molecular details of how they are coupled.

## Introduction

The maturation of nascent polypeptides relies on many factors that dynamically associate with the translating ribosome. These factors include modification enzymes, chaperones, targeting complexes, and protein translocons. While many fundamental aspects of protein translation are now understood in chemical detail (Voorhees and Ramakrishnan, 2013), far less is known about how these exogenous factors cooperate with the ribosome to facilitate nascent chain maturation.

A major class of proteins that rely extensively on ribosome-associated machinery are secreted and integral membrane proteins (Nyathi et al., 2013). In all organisms, a large proportion of these proteins are cotranslationally translocated across or inserted into the membrane. The exceptional prominence of this pathway in mammals is underscored by the original discovery of ribosomes as a characteristic feature of the endoplasmic reticulum membrane (Palade, 1955). Thus, understanding the nature of membrane-bound ribosomes and their role in secretory protein biosynthesis has been a long-standing goal in cell biology.

After targeting to the membrane (Egea et al., 2005), ribosomes synthesizing nascent secretory and membrane proteins dock at a universally conserved protein conducting channel (PCC), called the Sec61 complex in eukaryotes and the SecY complex in prokaryotes and archaea (Park and Rapoport, 2012). The PCC has two basic activities. First, it provides a conduit across the membrane through which hydrophilic polypeptides can be translocated. Second, it recognizes hydrophobic signal peptides and transmembrane domains and releases them laterally into the lipid bilayer.

These activities rely on binding partners that regulate PCC conformation and provide the driving force for vectorial translocation of the nascent polypeptide. The best characterized translocation partners are the ribosome and the prokaryote-specific ATPase SecA. Extensive functional and structural studies of the SecA-SecY posttranslational translocation system, in parallel with the cotranslational ribosome-Sec61 system, have coalesced into a general framework for protein translocation (Park and Rapoport, 2012).

Over the past two decades several crystal structures and cryo-EM reconstructions have led to numerous mechanistic insights into these events. High-resolution crystal structures of the large ribosomal subunit visualized the exit tunnel (Nissen et al., 2000), whose conserved conduit was shown to align with a bound Sec61 complex (Beckmann et al., 1997). While structural analysis of the prokaryotic ribosome and translation cycle progressed rapidly (Schmeing and Ramakrishnan, 2009), the lower resolution of parallel PCC structures (Menetret et al., 2000; Beckmann et al., 2001) posed a challenge to identifying changes in its conformation at different stages of translocation.

A major advance was the crystal structure of the archaeal SecYEβ complex (Van den Berg et al., 2004), which made several predictions about the nature and function of the translocation channel that were supported by later studies. The ten transmembrane segments of SecY are arranged in a pseudosymmetric orientation such that the two halves (formed by helices 1-5 and helices 6-10) surround an hourglass-shaped pore occluded by the plug domain. Six conserved hydrophobic residues from multiple surrounding transmembrane helices form a pore ring that lines the narrowest part of the channel and stabilize the conformation of the plug. Polypeptide translocation occurs through this central channel (Cannon et al., 2005), with the pore-ring residues contributing to maintenance of the membrane permeability barrier during translocation (Park and Rapoport, 2011).

Lateral egress of hydrophobic sequences from the SecY pore toward the membrane bilayer occurs through a lateral gate formed by the interface of helices 2 and 3 with helices 7 and 8. Crosslinking and cryo-EM studies support this as the site of signal peptide and transmembrane domain recognition and insertion (Plath et al., 1998; Park et al., 2014; Gogala et al., 2014; Mackinnon et al., 2014). Accordingly, impeding gate opening by crosslinking or mutagenesis impairs PCC function (Trueman et al., 2012; du Plessis et al., 2009). Together these studies identify the key structural elements of the Sec61/SecY channel that allow it to open across the membrane for translocation or toward the lipid bilayer for transmembrane domain insertion.

How these basic functions of the PCC are regulated by a translocation partner and the specific nascent polypeptide is incompletely understood. An X-ray structure of the SecA-SecY complex shows that interactions between the cytosolic loops of SecY with SecA induce a partial opening of the lateral gate and displaces the plug (Zimmer et al., 2008). These changes are thought to “prime” the channel for the ensuing polypeptide translocation. The analogous priming event with the ribosome has only been visualized at low-resolution (Park et al., 2014; Gogala et al., 2014), and thus is poorly defined. It is clear however, that ribosome interaction occurs via cytosolic loops between TM helices 6 and 7 (loop 6/7) and TM helices 8 and 9 (loop 8/9) (Ménétret et al., 2007; Ménétret et al., 2008). The precise nature of these interactions and how they affect key functional elements such as the plug or lateral gate remain unknown.

The subsequent stages of cotranslational translocation also remain to be resolved mechanistically. The various ribosome-PCC structures show that protein translocation is not accompanied by any major structural changes to the PCC (Menetret et al., 2000; Gogala et al., 2014). By contrast, engagement of a signal peptide or transmembrane domain opens the lateral gate to varying degrees (Park et al., 2014; Gogala et al., 2014), which may result in a conformation similar to that observed when a symmetry-related protein partially parted the lateral gate of SecY (Egea and Stroud, 2010). However, molecular insight into these regulatory events in a physiologic context require high-resolution structures of complexes engaged at different stages of the translocation pathway.

A number of recent technological advances in cryo-EM have permitted structure determination by single-particle analysis to unprecedented resolution (Bai et al., 2013; Li et al., 2013). These advances include the use of direct electron detectors, algorithms to correct for radiation-induced motion of particles, and improved computational methods for image processing and classification. Collectively, these advances have facilitated structure determination of the ribosome and associated factors, even when the relevant complex is present as a small percentage of a heterogeneous mixture (Fernández et al., 2013). In some instances, sufficient resolution can be achieved to build structures de novo and visualize the molecular details of key interactions (Amunts et al., 2014; Allegretti et al., 2014; Liao et al., 2013).

We reasoned that applying similar methods to a native membrane-bound ribosome solubilized from the endoplasmic reticulum could simultaneously provide mechanistic insights into both the mammalian ribosome and the associated translocation channel. At present, mammalian ribosome structures are limited to ∼5.4 Å resolution and have been bound to Stm1-like inactivating factors. Furthermore, features such as a native translating polypeptide and an A/P hybrid tRNA, characteristic of active elongation, have been difficult to trap in any system. A sample from an actively translating tissue, if sorted suitably, could overcome these limitations.

Similarly, a native sample of the PCC will also contain heterogeneity, due in part to the presence of associated factors such as the translocon-associated protein (TRAP) and oligosaccharyl transferase (OST) complexes (Ménétret et al., 2008); however, all particles should contain a single Sec61 complex. Furthermore, the linked nature of translation with translocation suggests that the translation state could indirectly inform on the status of the PCC. This could allow computational sorting of translating from idle PCCs on the basis of the ribosome. Thus, the recent methodological advances may allow sample heterogeneity to be transformed from an impediment to an advantage.

Here, we have determined structures of a porcine 80S ribosome-Sec61 complex in both an idle and translating state, determined to 3.4 and 3.9 Å resolution. These structures allow the detailed interpretation of the mammalian ribosome, the interaction between the Sec61 complex and the 60S subunit, and the conformational changes that occur to the channel during protein biogenesis.

## Results and Discussion

### Specimen Preparation and Characterization

**Figure S1:**
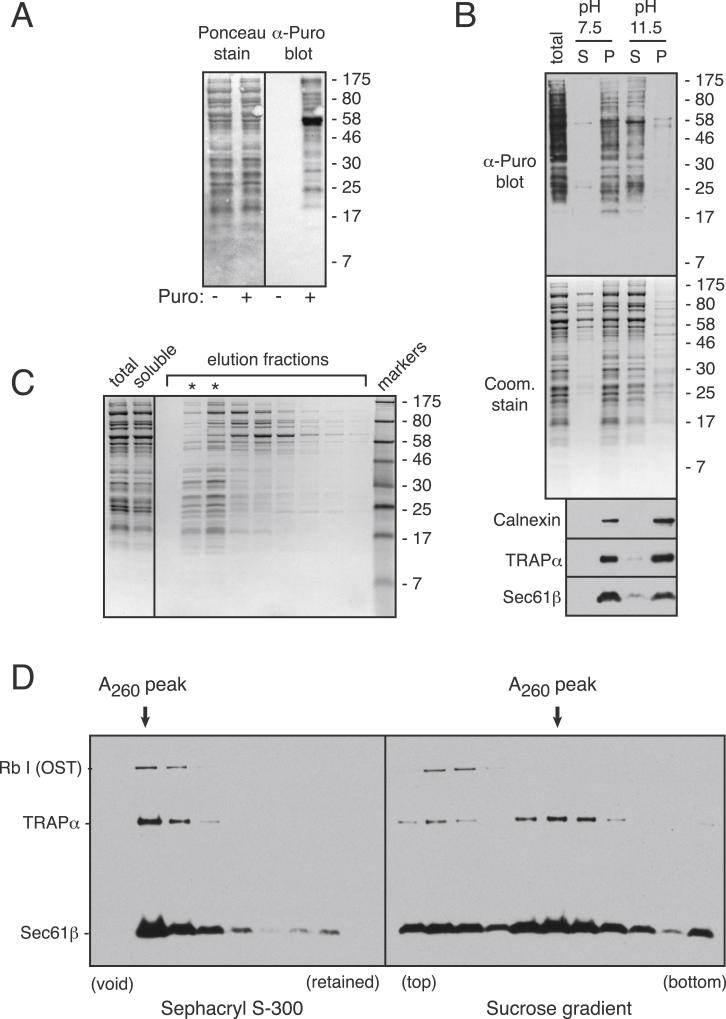
Biochemical Characterization of the Ribosome-Sec61 Sample, Related to Experimental Procedures (A) Immunoblot using anti-puromycin (DHSB anti-PYM 24As) of untreated and puromycin treated porcine pancreas microsomes. The ponceau stained blot is shown on the left. (B) Puromycin-treated pancreas microsomes (total) were extracted with buffer containing 0.1 M HEPES (pH 7.5), or 0.1 M Na_2_CO_3_ (pH 11.5), and subjected to ultracentrifugation (Fujiki et al., 1982). The supernatant (S) and membrane pellet (P) were analyzed by anti-puromycin immunoblot, coomassie staining, or immunoblot against the indicated integral membrane proteins. (C) Pancreas microsomes were solubilised with digitonin, clarified of insoluble material, and the extracted proteins separated by gel filtration using Sephacryl S-300 exactly as for samples prepared for cryo-EM analysis. Aliquots of the starting sample, soluble extract, and gel filtration fractions were analyzed by SDS-PAGE and coomassie staining. The asterisks show the ribosome-containing void fractions. (D) A digitonin-soluble extract prepared as in (C) was divided in two, fractionated by Sephacryl S-300 or 10%–50% sucrose gradient (as in Shao et al., 2013), and the fractions analyzed by immunoblotting for subunits of the OST, TRAP, and Sec61 complexes (antibodies characterized in Fons et al., 2003). The peak ribosomal fraction is indicated by absorbance at 260 nm. Note that most of OST and a portion of TRAP seem to dissociate during sucrose gradient fractionation.

The ribosome-translocon specimen was generated by fractionation of detergent-solubilized rough microsomes from porcine pancreas. Rough microsomes typically contain a mixture of actively translocating and quiescent ribosomes (Adelman et al., 1973). The presence of translationally active ribosomes in our microsomes was verified by labeling of their associated nascent polypeptides with puromycin (Figure S1A available online). Subsequent fractionation demonstrated that over 90% of puromycin-released nascent polypeptides were larger than ∼18 kD and cosedimented with the microsomes (Figure S1B). The vast majority of these polypeptides were efficiently extracted by alkaline sodium carbonate, a treatment that did not extract integral membrane proteins (Figure S1B). Thus, on average, the active translocon prior to solubilization contains a hydrophilic polypeptide passing through its central channel. In an attempt to capture these active ribosome-translocon complexes, we prepared our specimen with minimal time and manipulation between solubilization and freezing (Figures S1C and S1D).

### Structure Determination

**Figure S2:**
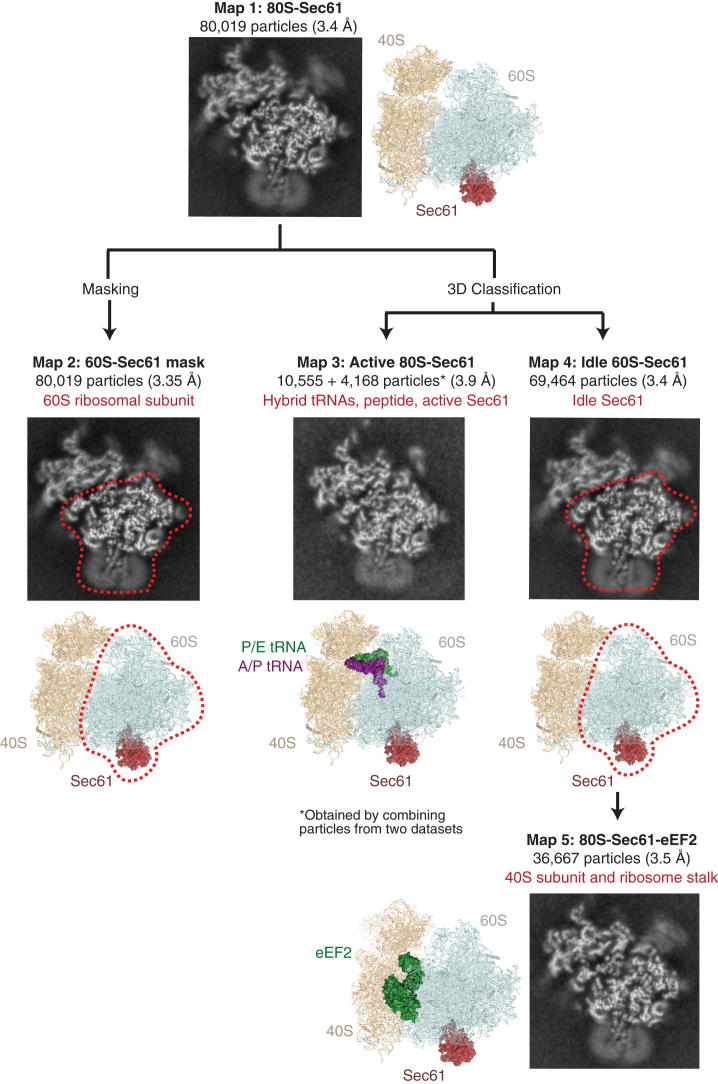
Refinement and 3D Classification Strategy, Related to Experimental Procedures Each class in the displayed flowchart shows a cross section image of the respective map, the derived model, any mask that was applied (red dashed line), and the structure (s) derived from that class (red text). The complete data set containing 80,019 particles was refined using RELION (Scheres, 2012a, 2012b), resulting in an initial reconstruction calculated to 3.4 Å resolution (Map 1). As the 40S subunit was in a variety of distinct conformations, a soft mask for the 60S subunit was used throughout the refinement procedure (Map 2). Although this only resulted in a modest nominal increase in resolution (3.35 Å), the observed density for the 60S subunit was improved compared to the complete refinement, and was used to build the 60S ribosomal proteins and RNA. In parallel, 3D classification of the entire data set identified 13% of particles containing hybrid A/P- and P/E-site tRNAs and a nascent peptide. In order to supplement this relatively small class, an additional data set was collected, resulting in a combined14,723 particles used to build the translating ribosome-Sec61 complex to 3.9 Å resolution (Map 3). The remaining 87% of particles (69,464) were used to model the idle ribosome-Sec61 complex, lacking a nascent chain and tRNAs, to 3.4 Å resolution (Map 4). Within this idle class, 36,667 particles containing eEF2, which stabilized both the stalk base proteins and the orientation of the 40S subunit relative to the 60S (Map 5). This class of particles was used to build a model for the 40S ribosomal proteins and RNA at 3.5 Å resolution. The remaining idle particles either contained eEF2 bound in different ratcheted conformations (19%) or were devoid of tRNAs or factors (22%). All reported resolutions are determined using the gold-standard Fourier Shell Correlation (FSC = 0.143) criterion (Scheres and Chen, 2012), and are shown in Figure S3.

Analysis of heterogeneous mixtures of particles visualized by cryo-EM is facilitated by improvements in image processing, in particular the use of maximum likelihood classification techniques (Scheres, 2010; Scheres, 2012b). Our initial data set contained 80,019 ribosomal particles. In silico classification of these particles (Figure S2) agrees with several aspects of its biochemical characterization. First, nearly all ribosomes contained a bound translocon, as classification of the final sample could not isolate any translocon-free ribosomes. Second, while the density for the area surrounding the translocon was heterogeneous due to a combination of accessory factors and the detergent-lipid micelle, very high occupancy was observed for the central Sec61 complex. Third, multiple classes of particles could be sorted based on the conformation of the ribosome and included translating and idle populations. The complete data set and individual classes were separately analyzed to extract their best features, which were incorporated into a composite model for the complete 80S-Sec61 complex.

**Figure 1:**
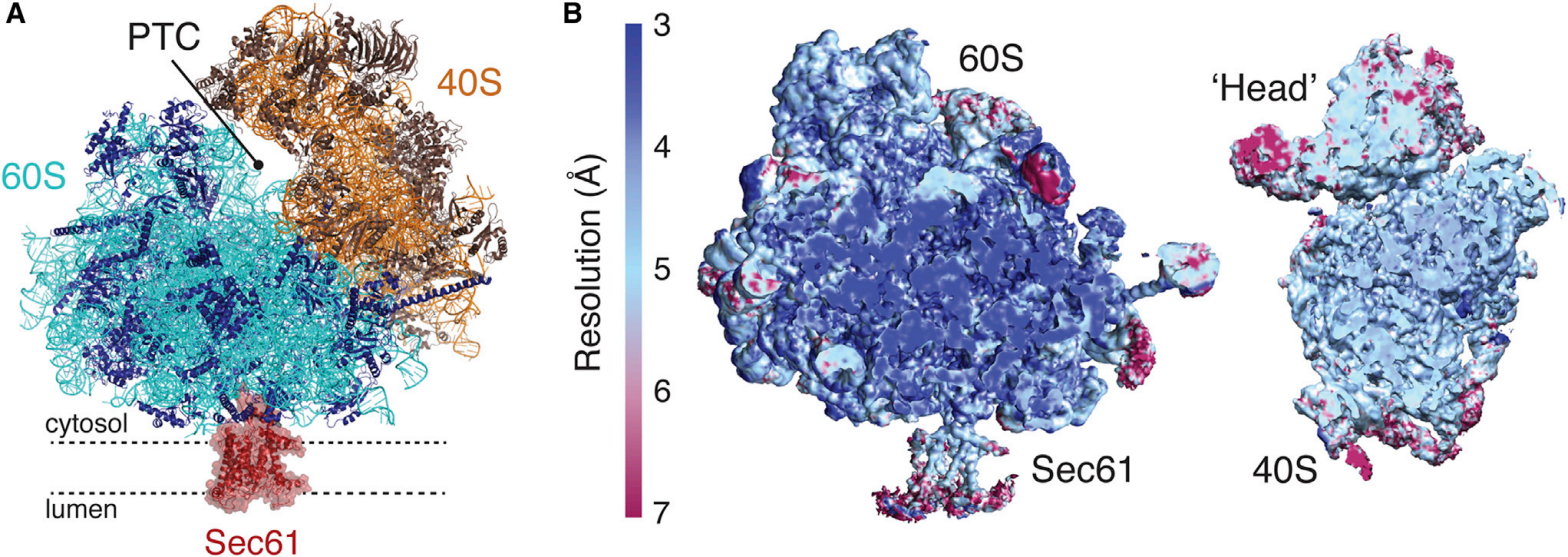
The Structure of a Mammalian Ribosome-Translocon Complex (A) Model of the idle 80S ribosome in complex with Sec61, shown in red. The color scheme shown here is used throughout the manuscript: 40S rRNA is displayed in orange, the 40S ribosomal proteins in brown, the 60S rRNA in cyan, and the 60S ribosomal proteins in dark blue. The region of the peptidyl transferase center (PTC) is indicated. (B) Cut view of the final unsharpened cryo-EM density map for both the idle 60S-Sec61 complex and the 40S subunit, colored by local resolution in Å (Kucukelbir et al., 2014). Also see Figure S3.

An initial reconstruction using the entire data set was calculated using a mask for the 60S subunit to avoid interference in the angular assignment by the heterogeneous conformation of the 40S. The resulting map, determined to 3.35 Å resolution, was used to build the ribosomal RNA and proteins of the 60S subunit. A distinctive class of ∼13% of particles contained two tRNAs bound in the A/P and P/E hybrid state. These particles were used to generate a 3.9 Å resolution map of the translating ribosome-translocon complex, within which density for the nascent polypeptide was observed throughout the ribosomal tunnel. The remaining 69,464 particles lacking tRNA and a nascent peptide were considered nontranslating ribosomes. This class was processed using a 60S mask to build the idle ribosome-Sec61 map at 3.4 Å resolution. Finally, this idle class was further subdivided by the degree of ribosomal ratcheting, and the presence or absence of the translational GTPase eEF2. One of these subclasses contained 36,667 particles and was used to produce a 3.5 Å resolution map used for building of the 40S ribosomal subunit and a well-ordered lateral stalk region. Thus, by leveraging major advances in both image detection and in silico analysis, a relatively small and heterogeneous data set could be used to build a near-complete atomic model of the mammalian 80S ribosome and high-resolution structures for the Sec61 complex bound to the translating and idle ribosome (see overview in Figure 1A). We will begin by presenting the structure of the 80S ribosome, followed by discussion of the Sec61 complex structure and its functional implications. Throughout this study, we use the new unified nomenclature for ribosomal proteins (see Table S1; Ban et al., 2014).

### An Atomic Model of the Mammalian Ribosome

**Figure S3:**
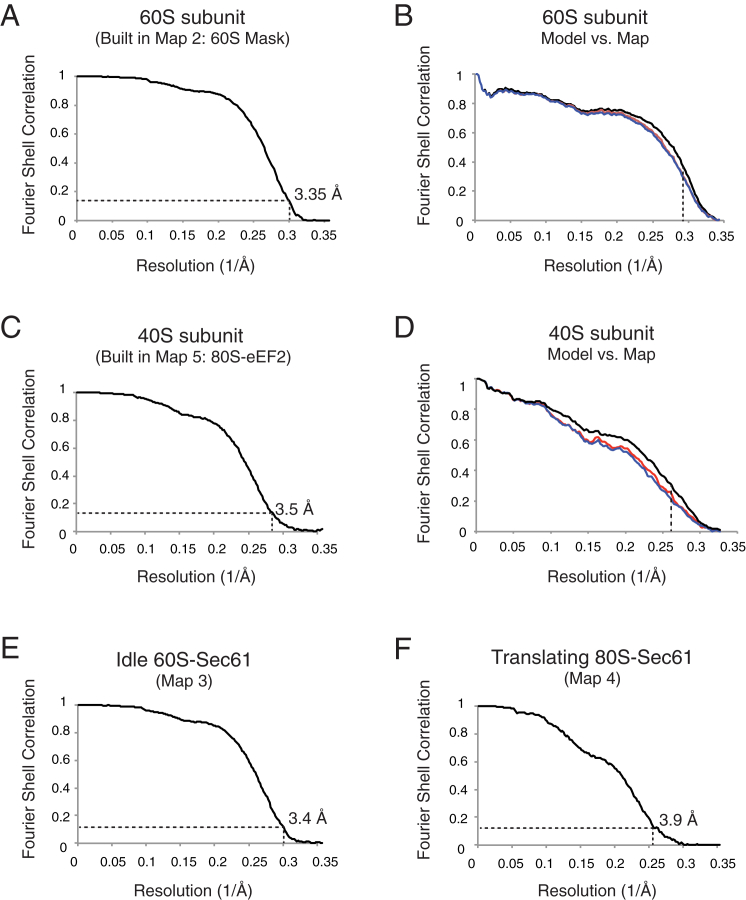
Map and Model Quality, Related to Figure 1 (A) Gold-standard Fourier Shell Correlation (FSC) curve for the map used for building of the 60S subunit (60S-mask, Map 2), where the resolution is demarcated using the FSC = 0.143 criterion. (B) FSC curves of the final model versus the full map used in A (black); of a model refined in the first of two independent halves of the map (red); and of that same model versus the second independent map, which was not used for refinement (blue). The vertical dashed line indicates the highest resolution used in these model refinements. (C and D) As in (A) and (B) but for the 40S subunit built using Map 5. The final refined models for both the 40S and 60S subunits were rigid-body fit into the density for each of the other classes of particles. (E and F) FSC curves for maps of the remaining classes.

The porcine ribosome described in this study was determined to an average resolution of 3.4 and 3.5 Å for the 60 and 40S, respectively (Figure S3, Table S2), as judged by the “gold-standard” Fourier Shell Correlation (FSC = 0.143) criterion (Scheres and Chen, 2012). Notably, much of the core of the 60S subunit is at 3.0 Å resolution or better (Figure 1B), while the head of the 40S subunit, given its inherent flexibility, is at somewhat lower resolution. The distal regions of several metazoan-specific rRNA expansion segments, such as ES27L, protrude from the ribosome and are presumably dynamic (Anger et al., 2013). As in the earlier study, these regions of rRNA were not visualized in our averaged maps. As the sample was prepared from an actively translating tissue, there was no evidence for binding of Stm1 or other sequestration factors that were observed in previous studies (Anger et al., 2013; Ben-Shem et al., 2011).

**Figure 2:**
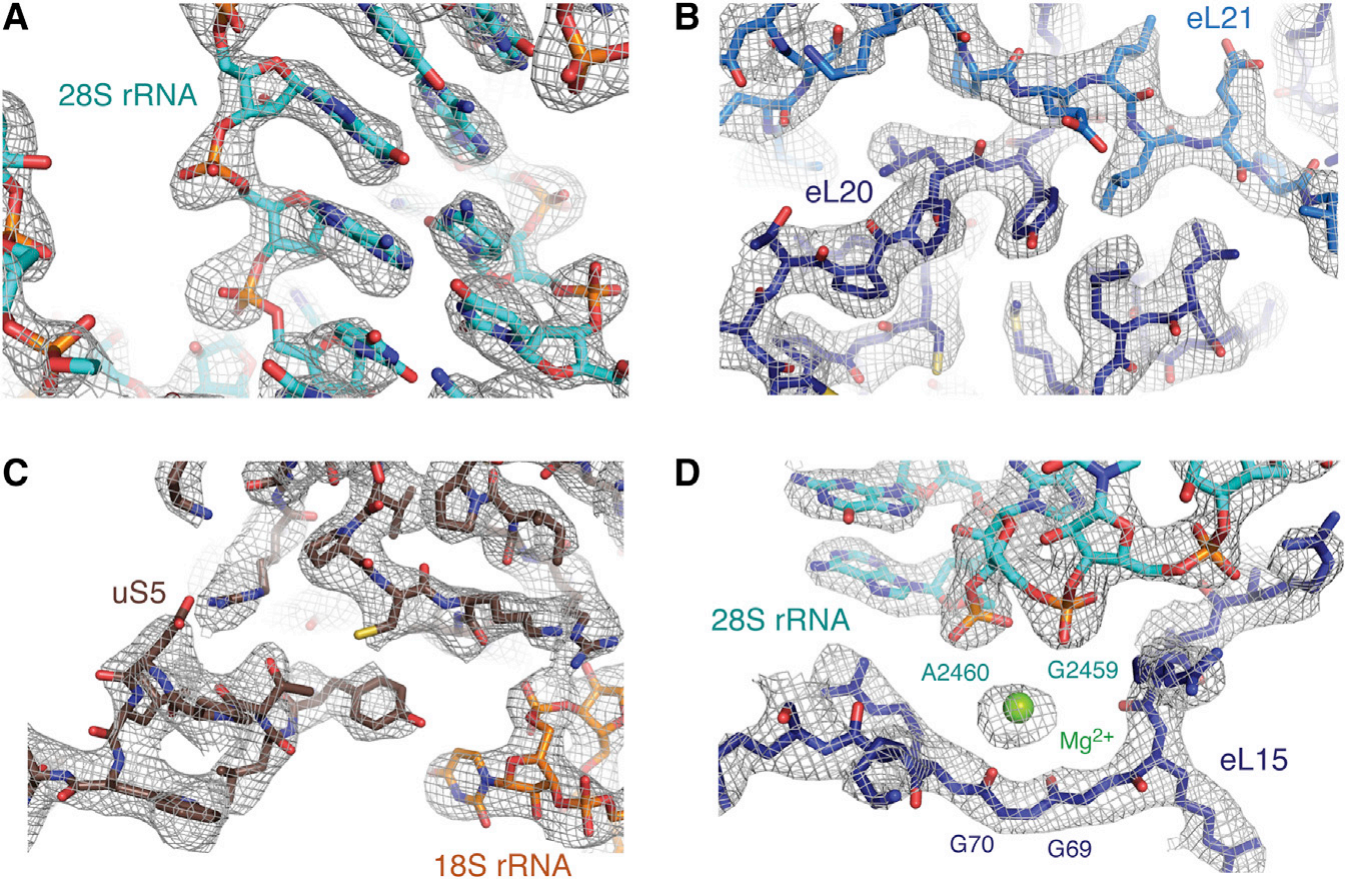
Representative Density for the Ribosomal Proteins and rRNA (A–D) Cryo-EM density for the 60S subunit and the body of the 40S was sufficient to allow unambiguous placement of rRNA bases (A, C, D) amino acid side chains (B, C, D), and many ions (D). Also see Figure S4.

**Figure S4:**
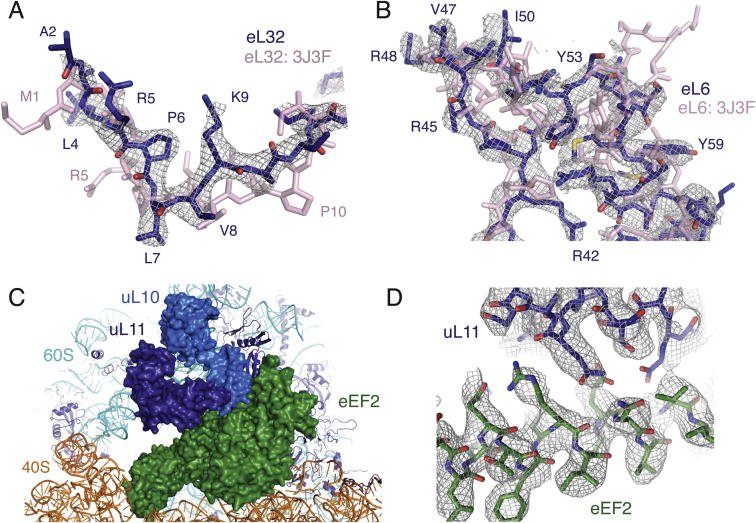
Examples of Revised and Newly Visible Ribosome Features, Related to Figure 2 (A) An example of a small change in registry in the N terminus of ribosomal protein eL32 (light pink; PDB ID 3J3F; Anger et al., 2013) that could be clearly visualized and remodelled (dark blue) using the improved density map. (B) An example of a mammalian specific expansion to ribosomal protein eL6 that could be modeled unambiguously using the new data. (C) Overview of binding of eEF2 in a half-ratcheted conformation, as calculated to 3.5 Å resolution using 36,667 particles. (D) Cryo-EM density for eEF2 and its interaction partners in the ribosome stalk were well-defined, allowing building of many side chains in the ribosomal proteins and GTPase. The chemical interaction between eEF2 and ribosomal proteins uL10 and uL11 could thus be visualized.

Using a recent model of the human ribosome generated at ∼5.4 Å resolution as a starting point (Anger et al., 2013), we have rebuilt each ribosomal protein and the rRNA, including many amino acid side chains, RNA bases, and over 100 Mg^2+^ ions (Figure 2). Our density map allowed de novo building of many regions that were previously approximated due to lower resolution (Figure S4A). Additional eukaryote-specific extensions of ribosomal proteins previously modeled by secondary structure predictions were also visible and built de novo (Figure S4B). The ribosome stalk was stabilized in the class of particles containing eEF2, which facilitated modeling at high resolution in this region (Figure S4C and S4D). As a result, we were able to build a near-complete 80S mammalian ribosome at atomic resolution. The marked improvement in the model is evident from the reduction of Ramachandran outliers within the ribosomal proteins from ∼13% (Anger et al., 2013) to ∼5.4% for the 60S subunit and ∼7.5% for the 40S (Table S2). The low percentage of Ramachandran outliers suggests the quality of our mammalian cryo-EM model is comparable to that of the seminal *S. cerevisiae* ribosome crystal structure determined to 3.0 Å resolution (Ben-Shem et al., 2011).

Unlike in bacteria, the eukaryotic ribosome relies on extensive protein-protein interactions (Ben-Shem et al., 2011; Anger et al., 2013), and the improved model presented here illustrates many of the detailed chemical interactions that stabilize the mammalian ribosome. For example, ribosomal proteins eL21 and uL30 together each contribute one strand of a β sheet, while stacking interactions are observed between a phenylalanine in eL20 and the 28S rRNA. Additionally, though eEF2 was bound in a nonphysiological state without P-site tRNA, its interactions with ribosomal proteins uL10 and uL11 can be observed at high resolution (Figures S4C and S4D). Given the high degree of confidence we now have in the model, and the extremely high sequence conservation of the ribosome in all mammals (Table S1), this structure will serve as a resource for future biochemical and structural experiments.

### Hybrid State tRNAs in an Actively Translating Ribosome

**Figure 3:**
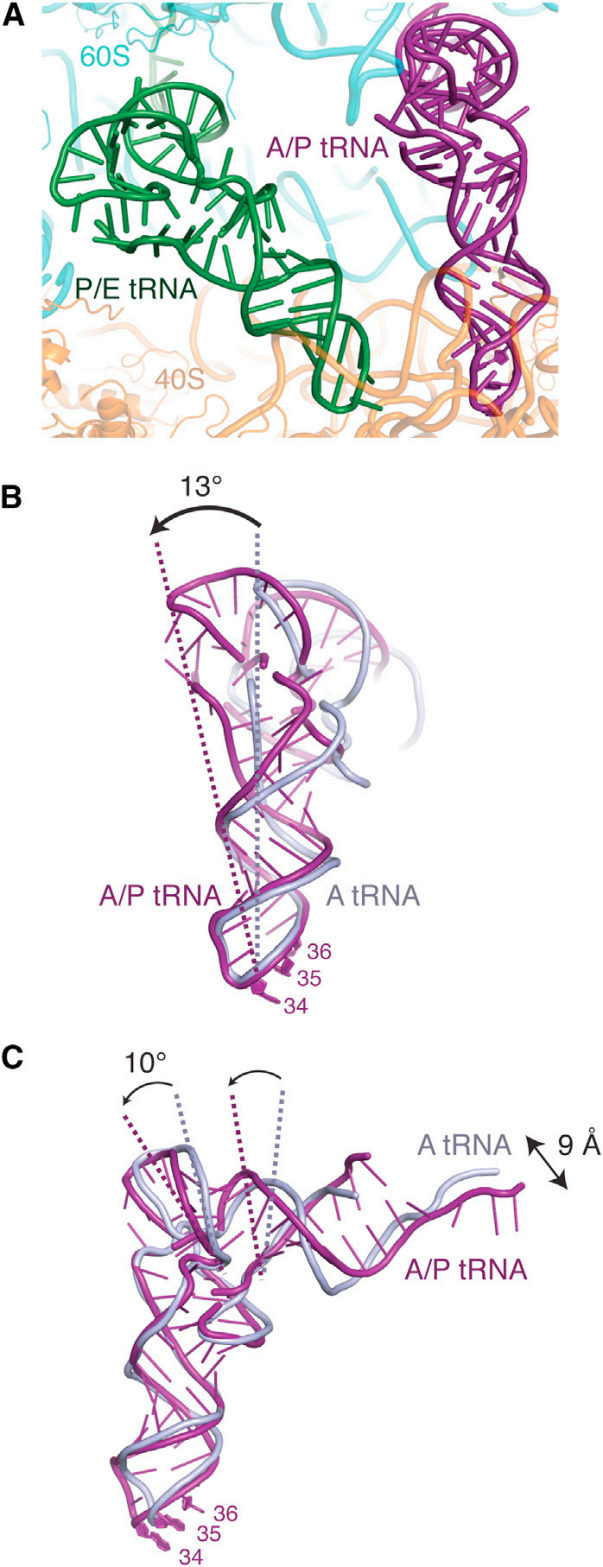
An A/P Hybrid State tRNA (A) Overview of the hybrid A/P (purple) and P/E tRNAs (green) visualized in the translating ribosome-Sec61 structure. (B and C) Adoption of the hybrid A/P conformation (purple) relative to the canonical A-site tRNA (gray) requires a ∼13° rotation in the backbone of the tRNA just above the anticodon stem loop, as well as a 10° rotation in the acceptor/T-stem stack and a 9 Å displacement of the 3′ tail.

The translating ribosome-translocon structure contained hybrid state A/P- and P/E-site tRNAs and a nascent polypeptide. The conformation of the P/E tRNA is similar to earlier reports (Tourigny et al., 2013; Dunkle et al., 2011) and stabilizes the L1 stalk inward. However, as previous reconstructions of an A/P tRNA were limited to ∼9 Å resolution (Agirrezabala et al., 2008; Julián et al., 2008), our structure represents the first high-resolution visualization of an A/P tRNA bound to the ribosome (Figure 3A). Though the sample contains a mixture of tRNA species, it was nevertheless possible to infer the global conformational changes required to adopt this hybrid conformation (Figures 3B and 3C).

In order to simultaneously bind the A-site mRNA codon and the 60S P site, the body of the tRNA must bend by ∼13° when compared to a canonical A-site tRNA (Voorhees et al., 2009). Notably, the CCA tail of the A/P tRNA does not superimpose with the 3′ end of a canonical P-site tRNA, presumably because in the hybrid state the 60S subunit is in a different orientation relative to the 40S. Thus, the hybrid A/P conformation is accomplished by an ∼9 Å displacement of the CCA tail, comparable to that observed in reconstructions of the bacterial complex (Agrawal et al., 2000), and by bending in two regions of the tRNA: the anticodon stem loop, and the acceptor/T-stem stack.

Similar regions have been implicated in binding of tRNAs to the ribosome in other noncanonical conformations (Schmeing et al., 2009). In particular, mutations in the anticodon stem loop have profound functional effects (Hirsh and Gold, 1971; Hirsh, 1971), as these mutations perturb the flexibility of the tRNA body and thus the energy required for adoption of these distorted conformations (Schmeing et al., 2011, 2009). Similarly, the A/P tRNA is undoubtedly a high-energy state stabilized by the presence of a nascent chain, which is discussed in further detail below. The instability of these intermediate tRNA conformations may favor movement of tRNAs and mRNA through the ribosome, facilitating translocation. Thus visualization of an A/P hybrid state further supports the notion that flexibility within the tRNA body must be precisely tuned to the requirements of the ribosome during protein synthesis.

### Overview of the Ribosome-Sec61 Structures

In addition to the high-resolution model of the ribosome presented above, analysis of the 80S-Sec61 complex afforded new insights into the role of Sec61 in translocation. The final models of a porcine ribosome-Sec61 complex in both an idle and translating state were determined to 3.4 and 3.9 Å resolution (Figures 1B, S2, and S3). Local resolution analysis of a cut away of the 60S subunit bound to Sec61 showed that the cytosolic regions of the idle Sec61 complex are at a similar resolution to the ribosome, and the resolution falls off only modestly toward the lumenal end (Figure 1B). Notably, the density threshold at which the ribosome was well resolved also afforded visualization of individual helices of the core Sec61 complex with almost no surrounding micelle or accessory factors. At a lower threshold, a large lumenal protrusion, which was previously identified as the TRAP complex (Ménétret et al., 2008) was observed together with the surrounding toroidal detergent-lipid micelle. Thus, these heterogeneous accessory components were either present at relatively low occupancy or highly flexible, with only the Sec61 complex well ordered in nearly every particle.

**Figure 4:**
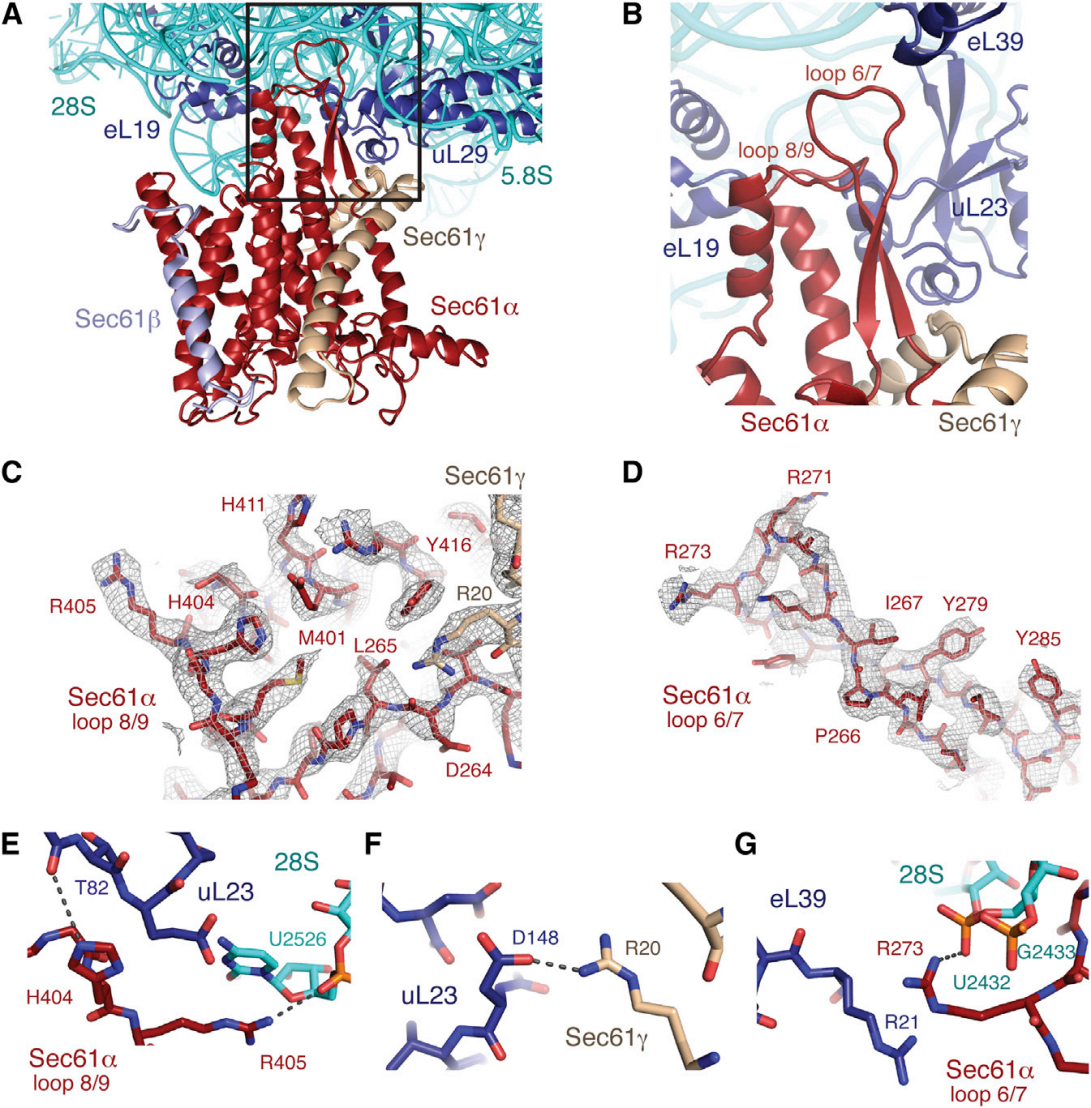
Interaction of Sec61 with the Ribosome (A) Overview of the region of the ribosome surrounding the Sec61 complex, including the cytosolic loops 6/7 and 8/9. Sec61α is displayed in red, γ in tan, and β in light blue. (B) Close-up of the cytosolic loops of Sec61 and the surrounding ribosomal proteins and RNA. (C and D) Representative density for the cytosolic loops of Sec61α, regions of Sec61γ, and their corresponding helices. (E) Hydrogen bonding interactions between residues H404 and R405 in loop 8/9 of Sec61α and ribosomal protein uL23 and the 28S rRNA. (F) Visualization of a salt bridge between R20 in the N-terminal helix of Sec61γ and D148 in υΛ23. (G) An arginine stack between residue R273 in loop 6/7 and R21 in eL39 is stabilized by interaction with the backbone of the 28S rRNA. See also Figure S5.

**Figure S5:**
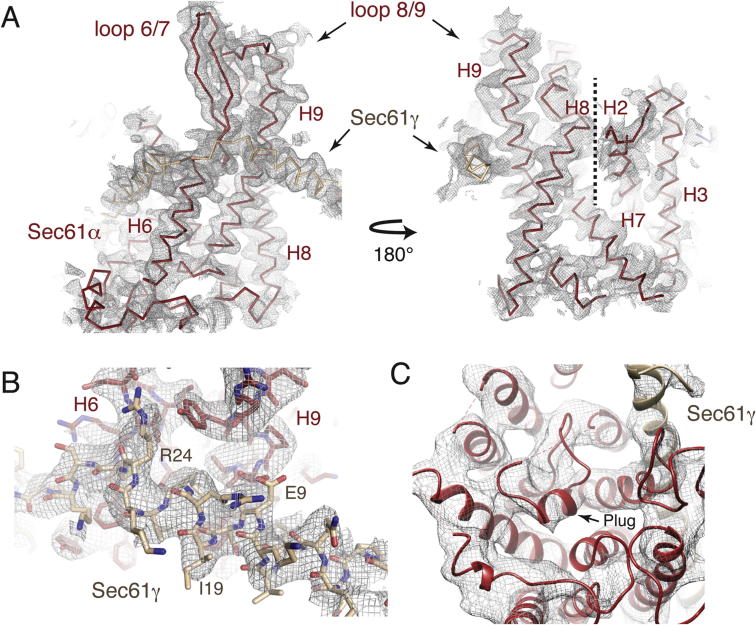
Density in Different Regions of the idle Sec61 Structure, Related to Figure 4 (A) Displayed here are two views of a density map filtered to 4.2 Å resolution that demonstrates the overall fit of the idle Sec61 channel. (B) Representative density for the N terminus of Sec61γ (tan), which was sufficiently ordered to allow placement of amino acid side chains. (C) Displayed is an unsharpened density map, in which the detergent micelle has been removed using Chimera (Goddard et al., 2007), visualized from the lumenal side of the channel to show placement of the backbone of the plug in the idle channel.

All three subunits of Sec61 are present, and have been unambiguously built into the density, including many amino acid side chains in the essential Sec61α and γ subunits (Figure 4, Figure S5). Notably, the two ribosome-associating cytoplasmic loops in Sec61α, between transmembrane helices 6 and 7 (loop 6/7) and transmembrane helices 8 and 9 (loop 8/9), have been built de novo (Figures 4C and 4D), as they have changed conformation compared to isolated crystal structures of SecY (Van den Berg et al., 2004; Tsukazaki et al., 2008). These loops were modeled only approximately in previous lower-resolution studies (Park et al., 2014; Gogala et al., 2014). Density for the nonessential Sec61β subunit is only visible in unsharpened maps displayed at low threshold, suggesting that it may be conformationally heterogeneous. We have therefore modeled only the backbone of the transmembrane helix of this subunit.

The overall architecture of the ribosome-bound mammalian Sec61 complex is similar to previously reported structures of the prokaryotic SecY determined by X-ray crystallography (Van den Berg et al., 2004). Earlier moderate resolution cryo-EM maps fit with homology models of the X-ray structures also show the same general architecture (Park et al., 2014; Gogala et al., 2014). However, given the significant improvement in resolution over these reconstructions, it is now possible to describe the atomic interactions of Sec61 with the ribosome and the nature of relatively subtle conformational changes that may occur within Sec61 during protein translocation.

### Interactions between the Ribosome and Sec61 Complex

Sec61 interacts with the ribosome primarily through the evolutionarily conserved loop 6/7 and loop 8/9 in the α subunit, as well as the N-terminal helix of Sec61γ (Figures 4A and 4B). The most extensive interaction surface is composed of loop 8/9 and Sec61γ, which together contact the backbone of the 28S rRNA and ribosomal proteins uL23 and eL29. Earlier structures implicated Sec61 interactions with uL29 (Becker et al., 2009). Although loop 6/7 packs against a loop of uL29, we could not observe specific contacts.

Specific interactions involve several conserved basic residues in loop 8/9, including His404, which interacts with Thr82 of uL23, and the universally conserved Arg405, which forms a stacking interaction with rRNA residue C2526 (Figure 4E). The hydroxyl group of Thr407 in helix 10, whose role in ribosome binding has not been previously predicted, is also within hydrogen bonding distance of the side chain of Asn36 of eL19. This may represent a conserved interaction, as the presence of a polar residue at position 407 has been evolutionarily retained. Finally, Arg20 of the γ subunit forms a salt bridge with Asp148 of uL23 (Figure 4F). These hydrogen bonding interactions stabilize the conformation of loop 8/9, and anchor the translocon at the exit tunnel. This observation is consistent with biochemical studies, which demonstrate that mutations to conserved residues in this loop cause a marked decrease in affinity of the translocon for the ribosome (Cheng et al., 2005).

Conversely, very few specific hydrogen-bonding interactions are observed for loop 6/7. Arg273 and Lys268 interact with phosphate oxygens within the 28S rRNA, while Arg273 appears to be stacking on Arg21 from protein eL39 (Figure 4G). Inverting the charge of Arg273 causes a severe growth defect in yeast, consistent with the observed interaction with the rRNA (Cheng et al., 2005). While it is clear that loop 6/7 is playing an important role in protein translocation due to its proximity to the ribosome, and its sequence conservation, the relatively small number of contacts suggest that it is unlikely to provide the primary stabilization of Sec61 to the ribosome. This is supported by the observation that although mutations within loop 6/7 cause profound defects in protein translocation and cell growth, they do not appear to affect ribosome binding (Cheng et al., 2005).

In all of the isolated crystal structures of SecY, cytosolic loops 6/7 and 8/9 are involved in a crystal contact (Van den Berg et al., 2004; Tsukazaki et al., 2008; Egea and Stroud, 2010) or interact with either a Fab or SecA (Tsukazaki et al., 2008; Zimmer et al., 2008). These loops appear to provide a flexible binding surface, likely due to their large number of charged and polar residues, which is exploited in both physiological and nonphysiological interactions.

### Conformation of Ribosome-Bound Sec61

**Figure 5:**
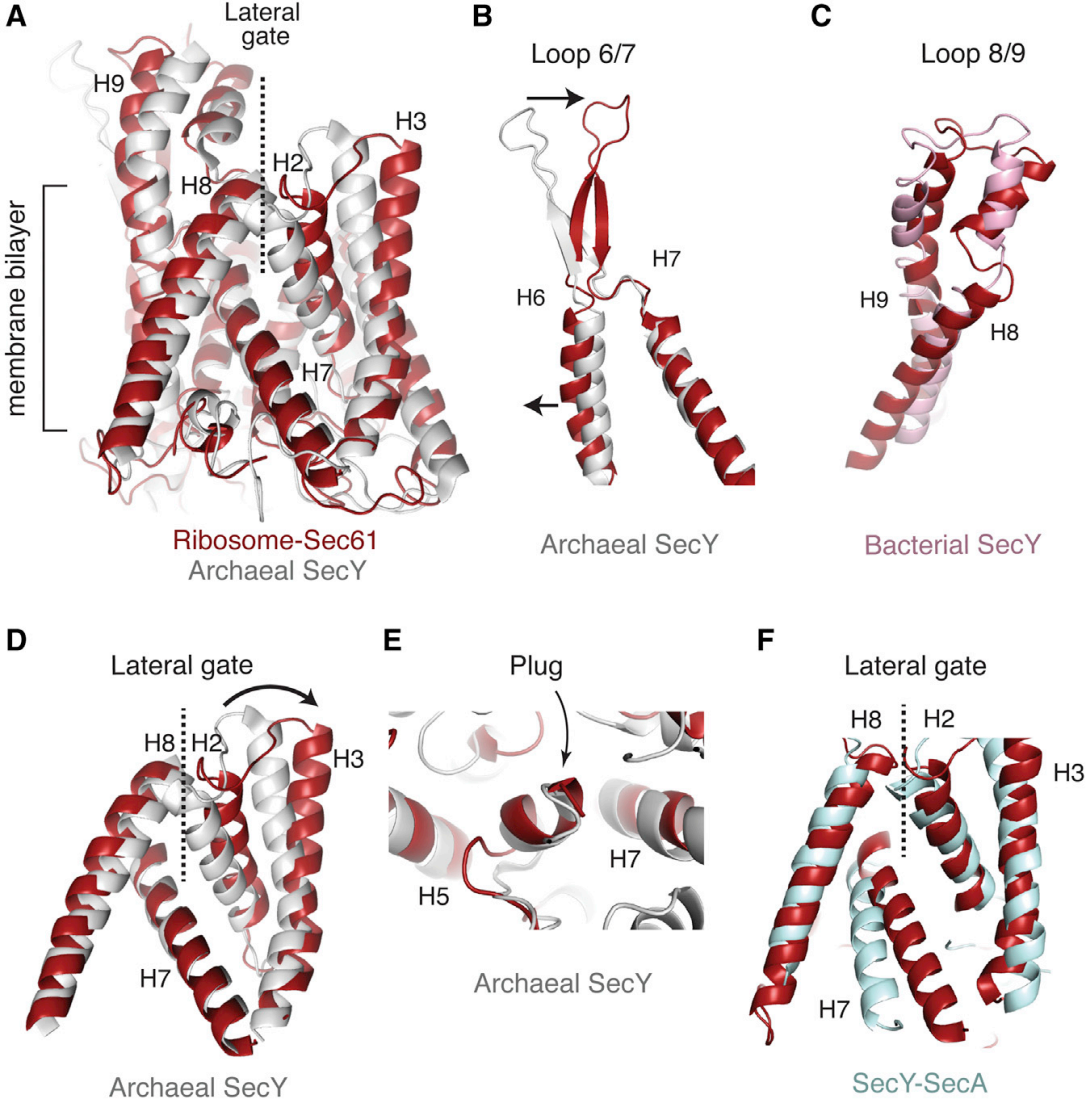
Conformation of Ribosome-Bound Sec61α (A) Overview of the lateral gate of the ribosome-bound Sec61α in red, compared to the isolated crystal structure of the archaeal SecY in gray (Park et al., 2014). (B) Cytosolic loop 6/7 shifts by 11 Å relative to the the archaeal SecY structure. (C) Cytosolic loop 8/9 shifts by 6 Å relative to the bacterial SecY structure shown in pink (Tsukazaki et al., 2008). The bacterial structure is used for comparison here because loop 8/9 is disordered in the archaeal structure. (D) Close-up of the lateral gate (helices 2 and 3 with helices 7 and 8), highlighting the opening of the cytosolic region between helices 8 and 2 in the ribosome-bound state. (E) Close-up of the plug region, which is unaltered in the ribosome-bound state. (F) Comparison of the lateral gate in the Sec61-ribosome structure relative to that observed in the SecY-SecA complex (light blue; Zimmer et al., 2008).

It has long been predicted that ribosome binding must prime the translocon to accept an incoming nascent chain. The idea is attractive because the channel must prepare to open toward the lumen or the membrane, requiring at least partial destabilization of the contacts that prevent access to these compartments. To gain insight into this priming reaction, we compared our idle ribosome-Sec61 structure to previous crystal structures from either archaea (Van den Berg et al., 2004) or bacteria (Tsukazaki et al., 2008). The implicit assumption in this comparison (Figure 5) is that the crystal structures approximate the preprimed quiescent state in the membrane. With this caveat in mind, we propose the following hypothesis for how ribosome binding could trigger a series of conformational changes that result in Sec61 priming.

In the ribosome-bound state, loop 6/7 is displaced relative to the isolated crystal structures, resulting in a rotation of the loop by 20–30 degrees (Figure 5B). Were the loop to remain in the conformation observed in the isolated structures, it would clash with either ribosomal protein uL29 or the 28S rRNA. It is likely that the extensive contacts between loop 8/9 and the ribosome, along with the clash with uL29 and the rRNA, constrain loop 6/7 into the observed conformation. Similarly, loop 8/9 is shifted by ∼6 Å, and the N terminus of the gamma subunit by ∼3 Å, compared to the isolated SecY in order to interact with the 28S rRNA and ribosomal proteins (Figure 5C).

The ribosome-constrained conformation of these loops transmits a small, but concerted distortion to their adjoining helices, which appears to be propagated helix to helix through the Sec61 channel. As the interhelical contacts in Sec61α are likely weakest at the lateral gate, these movements result in a slight opening between the cytosolic halves of helices 2 and 8 (Figure 5D). For example, residues G96 and T378 move from 4.4 Å apart in the isolated structure, to 11 Å apart on the ribosome. However, the intramembrane and lumenal portions of the lateral gate are largely unchanged and remain closed. An earlier model in which helix 8 bends substantially upon ribosome binding (Gogala et al., 2014) could not be supported by our higher-resolution map.

Furthermore, the plug is virtually unaltered from the conformation observed in the isolated structures (Figure 5E). The positions of helices surrounding the plug, which contribute pore-ring residues, also remain essentially unchanged. This suggests that the overall stability of the plug is not markedly altered by ribosome binding, although it is possible subtle differences in pore-ring interactions partially destabilize this region.

In total, these conformational changes may represent the priming of Sec61 upon binding of the ribosome. Though we cannot exclude the possibility that these movements are the result of sequence differences between archaea and mammals, this seems unlikely given the high degree of sequence conservation in the regions interacting with the ribosome and the interhelical contacts that change upon priming. Relative to the isolated crystal structures, the primed Sec61 has prepared for protein translocation by decreasing the activation energy required to open the lateral gate without altering the conformation or stability of the plug. Since targeting to the Sec61 complex is mediated by either a signal peptide or transmembrane domain, a cytosolically cracked lateral gate is ideally positioned to receive these forthcoming hydrophobic elements from SRP.

Indeed, a transmembrane domain stalled at a preinsertion state site specifically crosslinks to residues lining the cytosolic region of the lateral gate (Mackinnon et al., 2014). Insertion of a signal peptide or transmembrane domain into this site would further open the lateral gate, presumably destabilizing the plug. In this way, the channel’s opening toward the lumen would be coupled to successful recognition of a bona fide substrate.

Interestingly, movements of the lateral gate in Sec61, as described here, closely resemble those that occur upon binding of another translocation partner, SecA, to the cytosolic face of SecY (Figure 5F). As with the ribosome, SecA interactions with the cytosolic loops 6/7 and 8/9 also partially separate helix 8 and 2 at the lateral gate (Zimmer et al., 2008). These conformational changes may thus represent a universal mechanism for preparing the channel for translocation. However, the movements in the lateral gate with SecA are more exaggerated than with the ribosome: helix 7 shifts to increase the extent of lateral gate opening, while the plug is displaced toward the periplasm. Snapshots of the lateral gate and plug in a more open or closed form are also seen when SecY interacts with either an adjacent protein molecule (Egea and Stroud, 2010) or a Fab (Tsukazaki et al., 2008), respectively. Thus, the lateral gate interface would appear to be rather pliable and easily modulated by any number of physiologic or artificial interactions, particularly with the cytosolic loops.

### The Nascent Peptide in the Ribosomal Tunnel

**Figure 6:**
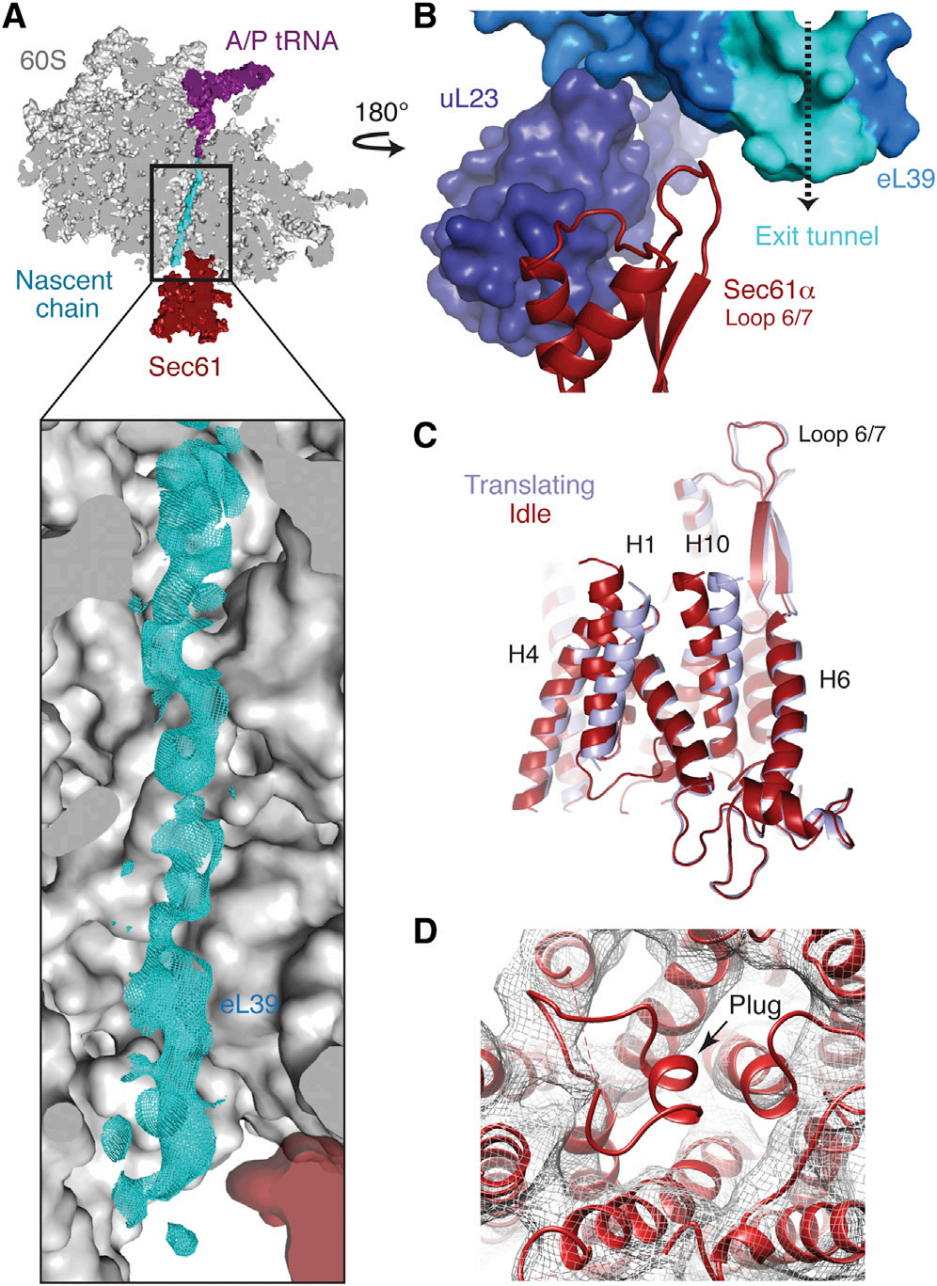
The Translating Ribosome-Sec61 Complex (A) Cryo-EM density within the ribosomal exit tunnel for the nascent peptide (cyan), which spans from the A/P tRNA to Sec61. The location of ribosomal protein eL39, which lines the exit tunnel, is indicated. (B) Ribosomal protein eL39 (bright blue) forms part of the exit tunnel (highlighted in cyan) and interacts with loop 6/7 of Sec61. Ribosomal protein uL23 (dark blue) contacts both eL39 and loop 8/9 of Sec61. (C) Comparison of the Sec61 channel structures bound to idle or translating ribosome, showing movements in helices 1 and 10, which may be important for allowing translocation of the nascent polypeptide. Also see Figure S6. (D) Rigid-body fitting of the idle Sec61 model (red) into the density for the translating Sec61-ribosome complex demonstrates that the plug is not visible in its canonical location. Displayed is an unsharpened map in which the disordered density for the detergent micelle has been removed using Chimera (Goddard et al., 2007).

Though the translationally active ribosome-Sec61 structure contains a heterogeneous mixture of translating polypeptides, it was possible to visualize near-continuous density in the ribosomal exit tunnel beginning at the tRNA and approaching the translocon (Figure 6A). No density in the exit tunnel was observed in the population of ribosomes without tRNAs. Through the majority of the tunnel, the observed density would be most consistent with an extended polypeptide chain. However, within the wider region of the ribosomal tunnel near the exit site, the density for the peptide broadens, suggesting that alpha-helix formation may be possible. As our sample contains an ensemble average of nascent chains, representing endogenous polypeptides, it suggests that all peptides follow a universal path through the ribosome, regardless of sequence or secondary structure tendency.

The density for the peptide first encounters Sec61 adjacent to loop 6/7, providing further evidence for the critical role this loop plays in protein translocation (Raden et al., 2000; Cheng et al., 2005). Several studies have hypothesized that there may be communication between the ribosomal tunnel and translocon to potentially prepare the channel for the handling of specific upcoming sequence domains (Berndt et al., 2009; Liao et al., 1997; Pool, 2009). As the rRNA lining the tunnel is relatively fixed, it has been proposed that such communication would involve the ribosomal proteins. The only protein that directly contacts Sec61 and partially lines the tunnel is eL39, which is positioned at the distal region of the tunnel (Figures 6A and 6B), where the peptide could begin to adopt secondary structure features. It is plausible that the conformation or hydrophobicity of the nascent peptide chain can be communicated via eL39 directly to loop 6/7 of the translocon (Figure 6B; see Figure 4G for detail). Alternatively, this communication could be transmitted via uL23, which forms extensive interactions with both eL39 and Sec61 at the surface of the ribosome (Figure 6B). The ability to visualize at near-atomic resolution both a defined nascent polypeptide and the Sec61-interacting ribosomal proteins surrounding the exit tunnel should allow these hypotheses to be directly tested.

### Structure of the Translating Ribosome-Sec61 Complex

Given the presence of the hybrid state tRNAs and nascent peptide, this class of particles clearly contains an actively translating ribosome-translocon complex. However, at a threshold at which nascent chain density is visible in the ribosomal tunnel, density was not observed within the Sec61 channel. One reason may be that upon exit from the ribosome, nascent chains have more conformational freedom inside a dynamic Sec61 than within the ribosomal tunnel. We cannot exclude the alternative possibility that nascent chains have slipped out of the Sec61 pore during sample preparation.

**Figure S6:**
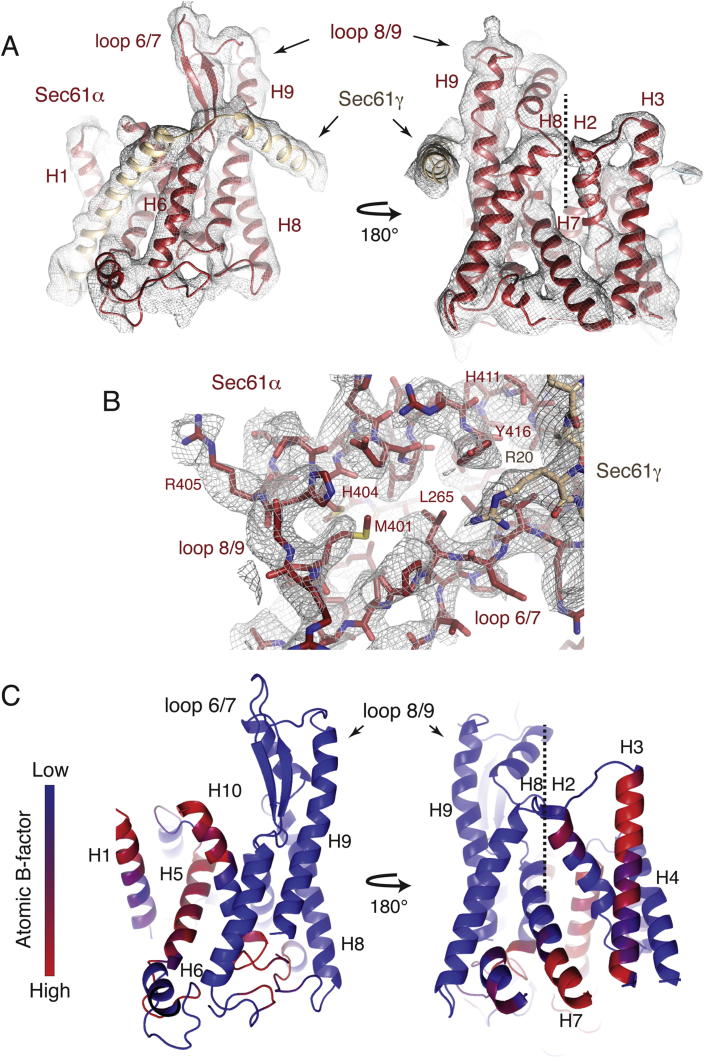
Density and Features of the Sec61 Structure Bound to the Translating Ribosome, Related to Figure 6 (A) Displayed is an unsharpened density map, in which the detergent micelle has been removed, to demonstrate the overall fit for the translating Sec61 channel. The same views as Figure S5A are shown for comparison. (B) Displayed is a sharpened map for the cytosolic loops of the translating ribosome-Sec61 structure. Though regions of the map are only visible to moderate resolution, areas of Sec61 that interact with the ribosome are well ordered and allow placement of amino acid side chains. (C) Model of the translating ribosome-bound Sec61α colored by atomic B-factor, with regions of lowest B-factor displayed in blue, and the highest in red. The same views as shown in (A) are displayed for comparison.

However, several lines of evidence suggest that most translating ribosome-Sec61 complexes in our sample contain a nascent chain within the Sec61 channel. First, the majority of polypeptides in this sample represent soluble proteins of at least ∼150 residues (Figure S1), a length more than sufficient to span the aligned conduits of the ribosome and Sec61 channel. Second, folded lumenal domains in most of these nascent chains would prevent back sliding through the pore during solubilization. Third, solubilization of pancreatic microsomes under conditions comparable to those used here retain nearly all endogenous nascent chains within the translocon (Matlack and Walter, 1995). Fourth, sample preparation after solubilization was very brief (<30 min) with minimal manipulations (Figures S1C and S1D), in contrast to the multistep purification that resulted in partial loss of nascent chains (Park et al., 2014). For these reasons, we provisionally interpret this structure as an “active” Sec61 channel in the discussion below; definitive proof must await a structure that permits direct nascent chain visualization. Though the resolution of this active Sec61 channel structure in many regions does not allow the same type of atomic level analysis as is possible for the idle translocon, it is still feasible to examine its main characteristics (Figures S6A and S6B).

In agreement with earlier studies (Gogala et al., 2014), the translocating state of Sec61 has no large-scale changes in its architecture (Figure 6C). Helices 2, 7, and 8 do not appear to have undergone substantial rearrangement, and the lateral gate is largely unchanged from the primed state. Additionally, helices 1 and 10 have shifted (Figure 6C), and the density for helix 3 is very weak (Figure S6A), suggesting it has become mobile. At a threshold where all the surrounding helices were visualized, density for the plug was no longer visible in the center of the channel (Figure 6D) and a continuous conduit now runs through Sec61α. The central pore was sufficiently large to house a model of an extended polypeptide without clashes.

While the plug’s canonical position was not occupied in the active state, we could not unambiguously assign it to an alternate location. It is possible the plug adopts a variety of conformations in this sample (given the heterogeneous sequences of translocating nascent chains) or becomes disordered to allow translocation. Given that the plug can be crosslinked to several disparate residues within an active SecY, it is likely dynamic once freed from its interactions with the pore ring. This flexibility may be facilitated by the observed movements in helix 1. In the static situation of a stalled nascent chain (Gogala et al., 2014), the plug may settle at its lowest energy state, perhaps explaining why it was apparently seen near its original location. However steric constraints would require at least a nominal shift in the plug to accommodate the nascent peptide within the central pore.

Although fewer particles for the active Sec61 complex led to a lower-resolution map than that for the idle complex, some areas are better resolved than others (Figures S6A and S6B). Helices 6-9, along with loops 6/7 and 8/9, display the highest resolution within the structure as judged by atomic B-factor (Figure S6C). This provides confidence in concluding that this part of Sec61 has few if any substantive conformational changes relative to the idle state. Thus, the C-terminal half of Sec61 effectively forms a stable platform for ribosome interaction.

By contrast, the density for helices 2-4 is significantly weaker than for either this same region in the idle Sec61 structure, or for helices 6-9 in the active structure (Figure S6). This observation strongly argues that the position of helices 2-4 in the active Sec61 is heterogeneous. Several nonmutually exclusive explanations are possible: (i) heterogeneous clients at different stages of transloction; (ii) different accessory proteins acting during translocation; and (iii) inherent flexibility in this region when the plug is displaced. Irrespective of the specific explanation (s), it would seem clear that helices 6-9 provide a ribosome-stabilized fulcrum, which allows movements within the remaining portion of the molecule to accommodate the nascent chain.

### Implications for Cotranslational Protein Translocation

**Figure 7:**
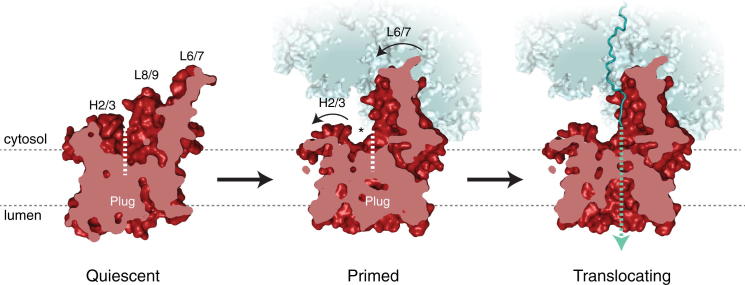
A Two-Step Model for Activation of Sec61 Displayed here is a cut-away view of the model for the Sec channel from the central pore toward the lateral gate (dashed line). In the quiescent state (left), approximated by a crystal structure of the archaeal SecY complex (Van den Berg et al., 2004), the Sec channel is closed to both the lumen and lipid bilayer. The channel becomes primed for protein translocation upon ribosome binding (middle), triggering conformational changes in Sec61 that crack the cytosolic side of the lateral gate (demarcated by an asterisk). The movements of helices 2 and 3 in this region may create an initial binding site for signal peptide recognition. Engagement of the lateral gate by the signal peptide would open the channel toward the membrane and initiate translocation (not depicted; Park et al., 2014). The translocating state of the active ribosome-Sec61 complex (right) contains a nascent polypeptide (teal) and is characterized by a dynamic plug domain and an open conduit between the cytosol and lumen (teal dotted line).

The structures described here help refine our understanding of several steps during cotranslational protein translocation and provide mechanistic insights into the two stages for fully activating the Sec61 channel (Figure 7). In the quiescent state presumably represented by the isolated crystal structure (Van den Berg et al., 2004), the channel is fully closed to both the lumen and lipid bilayer. The first stage of activation involves binding of the ribosome, which primes the channel by opening of the cytosolic side of the lateral gate, thereby decreasing the energetic barrier for translocation. The movement of helix 2, implicated as part of this priming reaction, may provide a hydrophobic docking site for the arriving signal peptide in this region. Importantly, this primed state leaves the channel largely closed to membrane and entirely closed to the ER lumen.

In the second stage of activation, a suitable substrate can now exploit the primed Sec61 by binding to and further opening the lateral gate. Signal peptide engagement at the lateral gate results in destabilization of the plug from the pore ring, either by sterically pushing the plug out of position, or by opening of the lateral gate, which shifts the helices surrounding the plug. Such a state appears to have been captured at low resolution in the *E. coli* system (Park et al., 2014). This model would rationalize why promiscuously targeted nonclients are rejected by Sec61, prior to gaining access to the lumenal environment (Jungnickel and Rapoport, 1995). The model would also explain how a small molecule that seems to bind near the plug can allosterically inhibit a signal sequence from successfully engaging Sec61 (Mackinnon et al., 2014).

Once the plug is destabilized, the translocating nascent chain can enter the channel, which sterically prevents the plug from adopting its steady-state conformation. A dynamic plug no longer stabilizes the surrounding helices at the central pore, permitting a more dynamic lateral gate. This flexibility may permit sampling of the lipid bilayer by the translocating nascent chain, thereby allowing suitably hydrophobic elements to insert in the membrane. This model for activation provides one explanation for why transmembrane segments within a multispanning membrane protein can be far less hydrophobic than those that engage the Sec61 channel de novo: the latter would need to fully open a nearly-closed lateral gate stabilized by the plug, while the former could take advantage of a gate made dynamic by plug displacement.

Both before and during translocation, a constant feature of the native ribosome-translocon complex is the substantial gap between the ribosome exit tunnel and Sec61. This gap has been consistently seen in many earlier structures (e.g., Ménétret et al., 2007) and presumably provides a site for release of cytosolic domains of membrane proteins. Secretory proteins are also accessible to the cytosol via this gap (Connolly et al., 1989; Hegde and Lingappa, 1996), and may be exploited for quality control of stalled or translocationally aborted nascent polypeptides (Zhou et al., 1998).

## Conclusions

The structures of the mammalian ribosome-Sec61 complex highlight the types of experiments made feasible by contemporary cryo-EM techniques. By studying a native, actively translating ribosome, it was possible to obtain high-resolution information for the conformation of an A/P tRNA and polypeptide within the exit tunnel, two states that are particularly challenging to capture using a reconstituted system. Furthermore, by using subsets of particles for different facets of the structure, otherwise dynamic elements such as the ribosome stalk could be visualized at high resolution. We anticipate that similar strategies will reveal the mammalian ribosome in various stages of its functional cycle, as well as translation-related regulatory events that impact human physiology (e.g., (Chen et al., 2014).

Analysis of a functionally heterogeneous mixture of particles also permitted direct comparisons of an idle and translating ribosome-Sec61 complex from the same sample. These structures allowed the detailed analysis of the interaction between Sec61 and the 60S subunit and the conformations acquired by the channel upon ribosome binding and protein translocation. These insights suggested a two-stage model for activation of the Sec61 channel, and provide a timeline for molecular changes leading to channel opening for peptide translocation or insertion. The challenge ahead will be to test these and other mechanistic hypotheses regarding the function of Sec61. Structures containing defined nascent peptides, stalled at intermediate stages of translocation, will allow us to precisely trace the sequence of events that accompany a nascent peptide’s transit from the ribosomal peptidyl transferase center into the ER lumen or membrane.

## Experimental Procedures

Additional details can be found online in Supplemental Information.

### Sample Preparation

Porcine pancreatic microsomes (Walter and Blobel, 1983) were solubilized in 1.75% digitonin, for 10 min on ice, clarified by centrifugation, and fractionated using Sephacryl S-300 resin in 50 mM HEPES (pH 7.5), 200 mM KoAc, 15 mM MgoAc, 1 mM DTT, and 0.25% digitonin. The void fraction was immediately processed for microscopy.

### Grid Preparation and Data Collection

Ribosome-Sec61 complexes at 40 nM were applied to glow-discharged holey carbon grids (Quantifoil R2/2), coated with a layer of amorphous carbon, and flash-cooled in liquid ethane using an FEI Vitrobot. Data were collected on an FEI Titan Krios operating at 300 kV, using FEI’s automated single particle acquisition software (EPU). Images were recorded using a back-thinned FEI Falcon II detector at a calibrated magnification of 104,478 (pixel size of 1.34 Å), using defocus values between 2.5–3.5 μm. Videos from the detector were recorded at a speed of 17 frames/s as previously described (Bai et al., 2013).

### Image Processing

Particle picking was performed using EMAN2 (Tang et al., 2007), contrast transfer function parameters were estimated using CTFFIND3 (Mindell and Grigorieff, 2003), and all 2D and 3D classifications and refinements were performed using RELION (Scheres, 2012a). The resulting density maps were corrected for the modulation transfer function (MTF) of the detector and sharpened as previously described (Rosenthal and Henderson, 2003; Amunts et al., 2014).

### Model Building and Refinement

The porcine 80S ribosome was built using the moderate resolution model for the human ribosome (Anger et al., 2013), while the Sec61 channel bound to both the idle and translating ribosome were built using the crystal structure of the archaeal SecY (Van den Berg et al., 2004) and the models of the canine Sec61 bound to the ribosome (Gogala et al., 2014). All models were built in COOT (Emsley et al., 2010), and refined using REFMAC v5.8 (Murshudov et al., 2011; Amunts et al., 2014). Secondary structure restraints for the Sec61 channel were generated in ProSMART (Nicholls et al., 2012). To test for overfitting, we performed a validation procedure similar to that described previously (Amunts et al., 2014). The final models for the 40S and 60S subunits were rigid-body fitted into the maps for the remaining classes, and refined. Figures were generated using Chimera (Goddard et al., 2007) and PyMOL (DeLano, 2006).

Extended Experimental Procedures **Sample Preparation**

Porcine pancreatic microsomes were prepared as previously described (Walter and Blobel, 1983), resuspended in membrane buffer (50 mM HEPES, 250 mM sucrose, 1 mM DTT) and flash frozen for long-term storage at −80°C. Microsome flotation and sucrose gradient experiments showed that all ribosomes in the preparation were membrane bound and primarily in polysomes (data not shown). Details of additional characterization are shown in Figure S1. To convert polysomes into monosomes, microsomes were adjusted to 1 mM CaCl_2_ and 150 U/ml micrococcal nuclease, incubated at 25°C for 7 min, adjusted to 2 mM EGTA, and flash frozen in single-use 50 ul aliquots. A 50 ul aliquot of nuclease-digested microsomes (at an A_280_ of 90) was adjusted with an equal volume of ice cold 2X solubiization buffer (3.5% digitonin, 100 mM HEPES [pH 7.5], 800 mM KOAc, 20 mM MgOAc_2_, 2 mM DTT) and incubated 10 min on ice. Earlier reports using similar solubilization conditions at both higher and lower salt observed no loss of nascent chains from the mammalian Sec61 channel as judged by protease protection assays (Matlack and Walter, 1995). Samples were spun for 15 min at 20,000 x g to remove insoluble material, and the solubilized material was fractionated by gravity flow over a 1 ml Sephacryl-S-300 column pre-equilibrated in column buffer (50 mM HEPES [pH 7.5], 200 mM KOAc, 15 mM MgOAc_2_, 1 mM DTT, and 0.25% digitonin). Roughly 100 ul fractions were manually collected and the void fraction containing ribosome-translocon complexes was identified by A_260_ measurements. The sample was centrifuged again as above to remove any potential aggregates before using immediately to prepare and freeze cryo-EM grids.

It is worth noting that we efficiently recovered nontranslating ribosome-Sec61 complexes despite using 400 mM KOAC during solubilization. Two reasons probably contributed to this high recovery: (i) solubilization and fractionation at a very high sample concentration, favoring an otherwise weak interaction; (ii) re-association of ribosomes with Sec61 when the salt concentration was reduced upon entering the gel filtration resin. Note for example that dissociation of translocon compoments was greater using sucrose gradient sedimentation than the more rapid Sephacryl-S-300 separation (Figure S1D). A second question is why a large proportion of our ribosomes contained no tRNA, some of which have eEF2. We do not know for certain, but differences from most earlier ribosome preparation protocols include isolating ribosomes from a microsomal subcellular fraction derived from a native tissue, the nuclease digestion reaction, and the specific conditions used for solubilisation and purification. Most earlier ribosome purification protocols are typically from total cell lysates, do not employ high detergent concentrations, often involve greater fractionation, and are bound to Stm1-like proteins.

**Grid Preparation and Data Collection**

Ribosome-Sec61 complexes were diluted in column buffer to a concentration of 40 nM, and were applied to glow-discharged holey carbon grids (Quantifoil R2/2) which had been coated with a ∼50–60 Å thick layer of continuous amorphous carbon. After application of 3 μl of sample, the grids were incubated at 4°C for 30 s, blotted for 9 s, and flash-cooled in liquid ethane using an FEI Vitrobot. Data were collected on an FEI Titan Krios operating at 300 kV, using FEI’s automated single particle acquisition software (EPU). Images were recorded using a back-thinned FEI Falcon II detector at a calibrated magnification of 104,478 (pixel size of 1.34 Å), using defocus values between 2.5–3.5 μm. Videos from the detector were recorded using a previously described system at a speed of 17 frames/s (Bai et al., 2013).

**Image Processing**

Semi-automated particle picking was performed using EMAN2 (Tang et al., 2007), which resulted in selection of 83,839 particles from 1,410 micrographs. A smaller second data set (referred to as data set 2 below) was collected later from another grid containing the same sample in order to increase the number of particles containing tRNAs and nascent chain. Data set 2 contained 726 micrographs that led to the selection of 37,061 particles. Contrast transfer function parameters were estimated using CTFFIND3 for both data sets (Mindell and Grigorieff, 2003), and any micrographs that showed evidence of astigmatism or drift were discarded at this stage. All 2D and 3D classifications and refinements were performed using RELION as described below (Scheres, 2012a, 2012b).

Unsupervised 2D class averaging was used to discard any nonribosome or defective particles, which resulted in a final data set of 80,019 80S particles, and an additional 36,696 particles from data set 2. Each data set was individually refined against a map of the *S. cerevisiae* ribosome filtered to 60 Å resolution, utilizing statistical movie processing in RELION as described previously (Bai et al., 2013).

As the 40S subunit was in several distinct conformations, a mask that included only the 60S subunit was used during refinement of the complete initial data set. This resulted in a final resolution of 3.35 Å using 80,019 particles for the 60S subunit as judged by the gold-standard FSC criterion (Scheres and Chen, 2012). In parallel, 3D classification of the initial 80,019 80S particles was performed using angular sampling of 1.8 degrees and local angular searches (using angles determined from the 3D refinement). Ten possible classes were allowed and resulted in the following. Class 4: representing ∼13% of the data set (10,555 particles), contained ribosomes in a ratcheted conformation with A/P- and P/E-site tRNAs and a nascent polypeptide in the ribosomal tunnel. Classes 6,7,9: three identical classes, together representing ∼46% of the data set (36,667 particles), contained ribosomes with eEF2 in a partially ratcheted orientation. Classes 2 and 3: together representing ∼19% of the data set, contained eEF2 bound to a ribosome in the canonical, unratcheted conformation. Classes 1,5,8,10: together comprising ∼22% of particles, contained empty ribosomes, without tRNAs or translation factors. Given the large percentage of particles that contain eEF2 in this sample (∼65%), weak density was observed for the factor in refinements using the complete data sets. However, continuous density for the factor could only be observed in refinements of appropriately grouped particles, described below. The apparent composition of the sample was similar for data set 2, from which 4,168 particles (∼11%) containing hybrid state tRNAs and a nascent peptide chain were combined with class 4 above to produce a larger set of particles for the translating ribosome-Sec61 structure.

Particles identified from 3D classification were combined according to biological state, and subjected to a final 3D refinement resulting in the density maps used for model building as described in Figure S2. The idle ribosome-Sec61 map was obtained using the 69,464 particles (Classes 1-3 and 5-10) that did not contain tRNAs or a nascent peptide, refined using a 60S mask to 3.4 Å resolution. The map of the translating ribosome-Sec61 complex, obtained by combining the first and second data sets, resulted in 14,723 particles that produced a 3.9 Å density map. The 40S subunit and the ribosomal stalk were best resolved in the 36,667 particles containing eEF2 (Classes 6,7,9) and the 40S subunit in a defined orientation, which extended to 3.5 Å resolution. All maps were corrected for the modulation transfer function (MTF) of the detector, and then sharpened using a negative B-factor (as described in Table S2), which was estimated using previously reported procedures (Rosenthal and Henderson, 2003). Local resolution of the final unsharpened maps was calculated using ResMap (Kucukelbir et al., 2014).

**Model Building and Refinement**

The porcine 80S ribosome was built using the moderate resolution model for the human ribosome (PDB IDs 3j3a, 3j3b, 3j3d, and 3j3f; Anger et al., 2013). Sec61 bound to both the idle and translating ribosome was built using the crystal structure of the archaeal SecY (PDB ID 1RH5; Van den Berg et al., 2004) and the low-resolution model of the canine Sec61 bound to the ribosome (PDB IDs 4CG7 and 4CG5; Gogala et al., 2014). All models were built in COOT (Emsley et al., 2010), and refined using REFMAC v5.8 (Murshudov et al., 2011) as previously described (Amunts et al., 2014). Registry and other errors to the ribosomal proteins were corrected manually, and each chain was refined individually against an appropriately cut map. Secondary structure restraints were generated in ProSMART (Nicholls et al., 2012), and nucleic acid base-pairing and stacking restraints were generated as before (Amunts et al., 2014) and were maintained throughout refinement to prevent overfitting. Ramachandran restraints were not applied, such that backbone dihedral angles could be used for subsequent validation of the refined models.

To test for overfitting, we performed a validation procedure similar to that described previously (Amunts et al., 2014). In brief, the final model was refined against an unsharpened density map calculated from only one half of the data using empirically determined chemical restraints. The resulting model was then used to calculate FSC curves for both halves of the data, one of which had been used during the refinement, and the other which had not (Figure S3). The two FSC curves nearly overlap, and we observe significant correlation beyond the resolution used for refinement (indicated by a vertical dashed line in Figures S3B and S3D), demonstrating that the model has predictive power and has not been overfitted. The models for the 60S and 40S subunits were then refined using these same restraints against the highest resolution sharpened maps for each subunit (Figure S2 Map2 and Map 5, respectively). The resulting models for the 60S subunit, the 40S body, and 40S head were individually rigid-body fitted into the maps for the remaining classes. All figures were generated using Chimera (Goddard et al., 2007) and PyMOL (DeLano, 2006).

## Author Contributions

R.M.V. and R.S.H. conceived the project. R.M.V. prepared and characterized samples, optimized them for EM analysis, and collected data. Particle selection, classification, and generation of initial maps were by R.M.V. with guidance from S.H.W.S. and I.S.F. Ribosome structure building and analysis was done by I.S.F. with help from R.M.V. Analysis of Sec61 structure was by R.M.V. with guidance from R.S.H. R.M.V. and R.S.H. wrote the paper with input from all authors.

## Accession Numbers

The EMDB accession numbers for the Cryo-EM density maps reported in this paper are 2644, 2646, 2649, and 2650. The Protein Data Bank accession numbers for the structures reported in this paper are 3J71, 3J72, 3J73, 3J74.

## Supplemental Information

Document S1. Tables S1 and S2

Document S2. Article plus Supplemental Information
