# Supplementary material for: Structure of the Mammalian Ribosome-Sec61 Complex to 3.4 Å Resolution
Source: Cell. 2014 Jun 19;157(7):1632–43. doi: 10.1016/j.cell.2014.05.024 (PMC4081569; doi:10.1016/j.cell.2014.05.024)
Supplement: Document S1. Tables S1 and S2 [file mmc1.pdf]

Table S1. Sequence Conservation between Pig and Human Ribosomes

Small Subunit Proteins		
**40S Subunit**	**'Old' Name**	**% Identity**
18S rRNA		99.4
eS1	S3A	98.0
uS2	SA	99.0
uS3	S3	100
uS4	S9	100
eS4	S4	100
uS5	S2	99.0
eS6	S6	100
uS7	S5	100
eS7	S7	100
uS8	S15A	100
eS8	S8	100
uS9	S16	100
uS10	S20	99.2
eS10	S10	99.4
uS11	S14	100
uS12	S23	99.3
eS12	S12	100
uS13	S18	100
uS14	S29	98.2
uS15	S13	100
uS17	S11	100
eS17	S17	99.3
uS19	S15	100
eS19	S19	100
eS21	S21	100
eS24	S24	99.2
eS25	S25	100
eS26	S26	100
eS27	S27	100
eS28	S28	100
eS30	S30	98.5
eS31	S27A	100
RACK1	RACK1	100

Large Subunit Proteins		
**60S Subunit**	**'Old' Name**	**% Identity**
Sec61α		100
Sec61β		100
Sec61γ		100
5S rRNA		100
5.8S rRNA		100
28S rRNA		94.1*
uL1	L10A	100
uL2	L8	100
uL3	L3	99.0
uL4	L4	96.8
uL5	L11	98.9
uL6	L9	100
eL6	L6	90.7
eL8	L7A	98.1
uL10	P0	99.7
uL11	L12	100
uL13	L13A	98.0
eL13	L13	96.7
uL14	L23	100
eL14	L14	90.7
uL15	L27A	97.3
eL15	L15	100
uL16	L10	99.1
uL18	L5	99.0
eL18	L18	98.4
eL19	L19	100
eL20	L18A	99.6
eL21	L21	100
uL22	L17	100
eL22	L22	99.2
uL23	L23A	100
uL24	L26	100
eL24	L24	100
eL27	L27	100
eL28	L28	97.8
uL29	L35	91.3
eL29	L29	87.0
uL30	L7	98.0
eL30	L30	100
eL31	L31	100
eL32	L32	100
eL33	L35A	100
eL34	L34	100
eL36	L36	100
eL37	L37	99.0
eL38	L38	100
eL39	L39	100
eL40	L40	100
eL41	L41	100
eL42	L36A	100
eL43	L37A	95.7

*The published porcine 28S sequence contains a ~550 nt gap in a non-conserved rRNA extension.

Table S2. Refinement and Model Statistics for the Final Models

**Data Collection**	**60S**	**40S**
Particles	80,019	36,667
Pixel size (Å)	1.34	1.34
Defocus range (mm)	2.5-3.5	2.5-3.5
Voltage (kV)	300	300
Electron dose (e/Å^2)	27	27
**Model Composition**		
Non-hydrogen atoms	136,883	75,528
Protein residues	6,461	4,844
RNA bases	5,181	1,869
Ions	130	36
**Refinement**		
Resolution for refinement (overall) (Å)	3.5 (3.35)	3.8 (3.5)
Map sharpening B-factor (Å^2)	-57.5	-59.8
Average B factor (Å^2)	37.0	65.7
R factor†	0.36	0.40
Fourier Shell Correlation (FSC)*	0.78	0.64
Rms deviations		
Bonds (Å)	0.015	0.011
Angles (°)	1.56	1.21
Ramachandran plot		
Favored (%)	94.6	92.5
Outliers (%)	5.4	7.5

† R factor = $\Sigma||F_{obs}|-||F_{calc}| / \Sigma|F_{obs}|$

* FSC=$\Sigma(F_{obs}F^{*}_{calc}) / \sqrt{(\Sigma|F_{obs}|^2 \Sigma|F_{calc}|^2)}$
